# The association of transcription factor Prox1 with the proliferation, migration, and invasion of lung cancer

**DOI:** 10.1515/biol-2021-0056

**Published:** 2021-06-19

**Authors:** Xinxin Hao, Wenting Luo, Xueshan Qiu

**Affiliations:** Department of Pathology, The First Affiliated Hospital and College of Basic Medical Sciences, China Medical University, Shenyang 110001, China; Department of Blood Transfusion, Shengjing Hospital of China Medical University, Shenyang 110004, China; Key Laboratory of Health Ministry for Congenital Malformation, Shengjing Hospital of China Medical University, Shenyang 110004, China

**Keywords:** lung cancer, transcription factor Prox1, Rho family, migration, invasion

## Abstract

**Background:**

The current study investigates the effect of transcription factor Prox1 on the proliferation, migration, and invasion ability of lung cancer.

**Methods:**

Lung cancer cell lines (A549 and H446 cells) were transfected with Prox1NAD and siRNA, respectively. Thus, the A549 and H446 cells overexpressed Prox1 after transfection of Prox1NAD plasmids, and A549 and H446 cells have low expression of Prox1 after transfection with siRNA. Reverse transcriptase quantitative PCR and western blot analyses were used to detect Prox1 mRNA and protein expression in cells. Plate clone formation experiments and MTT experiments were used to detect cell proliferation. Western blot was used to detect the expression of Rho family-related proteins in cells.

**Results:**

Compared to untransfected wild-type A549 and H446 that served as blank controls, the expression level of Prox1mRNA and protein in A549 and H446 cells overexpressing Prox1 after plasmid transfection was high, while the expression level of Prox1mRNA and protein in A549 and H446 cells with low expression of Prox1 after siRNA transfection was low. With the increase of Prox1 expression, the expression of RhoA and RhoC increased, while the expression of RhoB decreased.

**Conclusion:**

The finding of this study may provide a new approach for the treatment of lung cancer using targeted gene therapy.

## 1 Introduction

Lung cancer is the most common primary malignancy of the lung, and it is currently the most common type of highly heterogeneous malignancy in the world [1,2]. According to relevant clinical statistics, global lung cancer incidence has been on the rise since the late twentieth century. The global average incidence accounts for 7–20% of various malignant tumor diseases throughout the body. In recent years, the 5-year survival rate of lung cancer in China is only 16.1% [3]. According to the 2009 data released by the National Cancer Center and the Bureau of Disease Control and Prevention of the Ministry of Health in 2012, lung cancer ranked first in the global cancer incidence [4]. Although in the past few decades, progress has been made in various treatment methods, such as drug therapy, radiation therapy, and targeted gene therapy, and the overall survival rate of lung cancer has improved, lung cancer is still the leading cause of health threats, and so the research on the pathogenesis of lung cancer is still a hot spot [5,6]. The occurrence of lung cancer is a multistep, multifactor, multistage development process, which involves the expression of a series of oncogenes and related metastatic genes [7–9]. Prox1 is a transcription regulator capable of regulating cell differentiation and development, which is essential in the development process of human liver and neurons [10–12]. Current research has shown that Prox1 is overexpressed in tumor tissues, such as kidney cancer and gastric cancer, while downregulated in tumor tissues, such as primary liver cancer, indicating that it may play different roles in different tumor tissues [11]. Prox1 is implicated in the growth and progression of cancer and has been linked in different cancer types to both tumor-suppressive and oncogenic properties. Nevertheless, the exact mechanisms by which Prox1 controls cancer cell proliferation, migration, and invasion are largely unclear [12]. A previous study showed that Prox1 expression in small-cell lung cancer cell line is high and can be reduced with shRNA lentivirus, thereby reducing the cell proliferation rate [13]. Therefore, to further explore the molecular mechanism of Proxl in tumorigenesis, especially the closely related lymphangiogenesis mechanism, lymphatic metastasis pathways and related molecular pathways are investigated. Moreover, to study the cure of its therapeutic targets for diseases, especially tumor diseases. The prognosis is of great significance and has good clinical application prospects. Previous studies have reported that Prox1 can positively regulate the growth, migration, and invasion of renal cancer cells. Prox1 expression levels are correlated with cancer progression and prognosis. For instance, high Prox1 expression in human colon and esophageal cancer tissues is correlated with poor prognosis [14,15]. Tumors with high Prox1 expression have a worse prognosis. The specific knockdown of the Prox1 gene by in vitro RNA interference strongly reduces cell growth, whereas overexpression of Prox1 can significantly promote tumor proliferation [16].

In the current study, we transfected plasmid overexpressing Prox1 and plasmid siRNA silencing Prox1 to study the role of Prox1 in the proliferation, invasion, and migration of A549 and H446 lung cancer cells. The findings of the current study will provide a new approach for the treatment of lung cancer using targeted gene therapy.

## 2 Methods

### 2.1 Experimental material

#### 2.1.1 Cell lines

Human lung adenocarcinoma cell line A549 and non-human lung adenocarcinoma cell line H446 were purchased from the cell bank of the Chinese Academy of Sciences in Shanghai.

#### 2.1.2 Related reagents and antibodies

Trizol reagent was purchased from Invitrogen. RT-PCR kit was purchased from Beijing Quanshijin Biotechnology Co., Ltd. Real-time PCR kit was purchased from Takara Bio. Transwell cell was purchased from Corning Costar Corporation, USA. Matrigel matrix gelatin was purchased from BD. Prox1 antibody from was purchased from Abcam, UK. RhoA, RhoB, and RhoC antibodies were purchased from Proteintech, USA. β-Actin antibody was purchased from Cell Signaling Technology, USA.

### 2.2 Experimental method

#### 2.2.1 Cell culture and transient transfection

A549 and H446 cells were cultured in a DMEM and 1640 medium containing 10% fetal bovine serum in an incubator at 37°C and 5% CO_2_ saturated humidity for logarithmic growth. Logarithmically growing cells were seeded onto a 6-well plate for 24 h according to 15,000 cells/well, and the growth rate of cells was observed for transfection. Then, Prox1DNA and siRNA were added into the HiPerFect transfection reagent and Attractene transfection reagent, respectively, and mixed with double medium-free in two EP tubes. After 10–15 min, Prox1DNA and siRNA were washed with PBS twice and added with medium containing 10% serum. The mixed plasmids were evenly dropped into the 6-well plate and incubated overnight. RNA and protein were extracted from cells after 24 and 48 h, respectively.

#### 2.2.2 Plate colony formation assay

Cells from different treatment groups were transfected for 24 h and then digested and passaged routinely to make cell suspensions to disperse the cells fully. Cells were counted, and the concentration was adjusted. A total of 500 cells per well were mixed into 2 mL medium, then dropped into the 6-well plate evenly, and dispersed by gently shaking. They were placed in an incubator for 14 days, and the medium was changed in time according to the pH change of culture solution. When visible colonies appeared, cells were grown and cultivated, the medium was discarded, and then washed carefully with PBS. Air drying after methanol fixation and hematoxylin staining for 10 min was carried out. When the tap water turned blue, plates were observed under the microscope.

#### 2.2.3 MTT assay

Cell inoculation: Upon entering the logarithmic growth phase, the cells were suspended with the medium containing 10% fetal bovine serum after digestion. The cell concentration was adjusted to 4 × 10^3^ cells/mL, 100 µL cell suspension per well was inoculated into a 96-well plate, and the control group was set up. Color reaction: 20 µL of MTT solution with a concentration of 5 mg/mL was added to each well, avoiding the light. After incubation at 37°C for 4 h, the old medium was discarded, and 150 µL of DMSO was added to each well and shaken on the shaker for 5 min. Colorimetric detection: The absorption value of each well was measured by 490 nm Thermo Scientific™ enzyme-labeling measuring instrument.

#### 2.2.4 Western blot hybridization

The 6-well plate was taken out from the incubator, and the precooling cracking buffer was added, sheared, ultrasound homogenated, and centrifuged at 4°C, 1,200 rpm for 30 min. The supernatant was collected as the total protein, the protein concentration was measured, the protein buffer was added according to the concentration, and the sample in boiling water was boiled for protein denaturation and stored at −20°C. 10% SDS-PAGE electrophoresis, 20% methanol transfer for 90 min, buffer washing membrane for 15 min three times, and 5% skimmed milk powder blocking non-specific antigen for 2 h were carried out, after which the membrane was washed with a buffer for 15 min three times and incubated with Prox1 primary antibody (1:800) at 4°C overnight. The membrane was washed with a buffer for 15 min three times and incubated with alkaline phosphatase labeled second antibody (1:2,000) at room temperature for 2 h. For ECL color reaction, the clear chocolate brown band on NC film was positive, and a SYSTEM GelDoc go gel imaging system (Bio-Rad, USA) quantitatively analyzed the grey value scanned by the color band.

#### 2.2.5 Reverse transcriptase quantitative RNA polymerase chain reaction

For total RNA extraction, 1 mL of Trizol solution and 200 μL of chloroform were added to the cells. After shaking and mixing, centrifugation at 4°C, 12,000 rpm for 15 min was carried out. The supernatant was transferred to the new EP tube, an equal amount of isopropanol was added, mixed well, and left at room temperature for 10 min. After 15 min of centrifugation, the supernatant was discarded, precooled 75% alcohol was added, and the probe was mixed. Following yet another 10 min centrifugation, the supernatant was discarded, then precooled ethanol was added, shocked, and mixed. After centrifuging for 10 more minutes, the supernatant was discarded and dried, following which RNase-free water was added and incubated for 10 min. The total RNA absorbance value was measured using an ultraviolet spectrophotometer to identify the purity of RNA. The RNA was stored at 80°C for later use. Agarose gel electrophoresis: Total RNA from cells was extracted and reacted according to the method of Easy-RT-PCR kit. PCR products were detected by agarose gel electrophoresis, and the SYSTEM GelDoc go gel imaging analysis system (Bio-Rad) was used to perform a semi-quantitative analysis. Total RNA was extracted from cells and reacted according to the method of SYBR PrimeScript RT-PCR kit. Three wells were set for all reactions.

#### 2.2.6 Transwell migration experiment

Migration experiment: Prox1 plasmid, pcdna3.0 plasmid or siRNA, NC A549, SPC, and H446 cells were transfected in 24-well plate for Transwell. The cells were sterilized by UV irradiation, and the upper surface was hydrated by a serum-free medium. The hydrated Transwell cells were put into a 24-well culture plate. Outside the cell, 500 µl of 10% fetal bovine serum culture solution was added, and inside the cell, 100 µl of transfected cell suspension was added. The cell number was 2.5 × 10^5^/well, and no fetal bovine serum was added to the culture solution. After 24 h of routine culture, Transwell cell was taken out, washed with PBS, and the upper cells of the microporous membrane were carefully wiped off with cotton swabs, fixed with methanol, and stained with hematoxylin solution. The number of cells moved to the lower layer of the microporous membrane was counted under a fluorescence microscope. Invasion experiment: The cells were sterilized by UV radiation, and the hydrated Transwell cell was placed in a 24-well culture plate. Matrix gelatin was mixed with double medium-free at a ratio of 1:3 and spread evenly in the upper chamber at 20 µL per well. After gelation in the incubator for 4 h, 500 µL of culture solution containing 10% fetal bovine serum was added outside the cell, and 100 µL of transfected cell suspension was added into the cell; the cell number was 2.5 × 10^5^/well. After 24 h of routine culture, Transwell cell was taken out, washed with PBS, and the upper cells of the microporous membrane were carefully wiped off with cotton swabs, fixed with methanol, and stained with hematoxylin solution. The number of cells that moved to the lower layer of the microporous membrane was counted under a fluorescence microscope. Each sample counted 6–10 fields of view, and the experiment was repeated three times.

### 2.3 Statistical analysis

**Figure 1:**
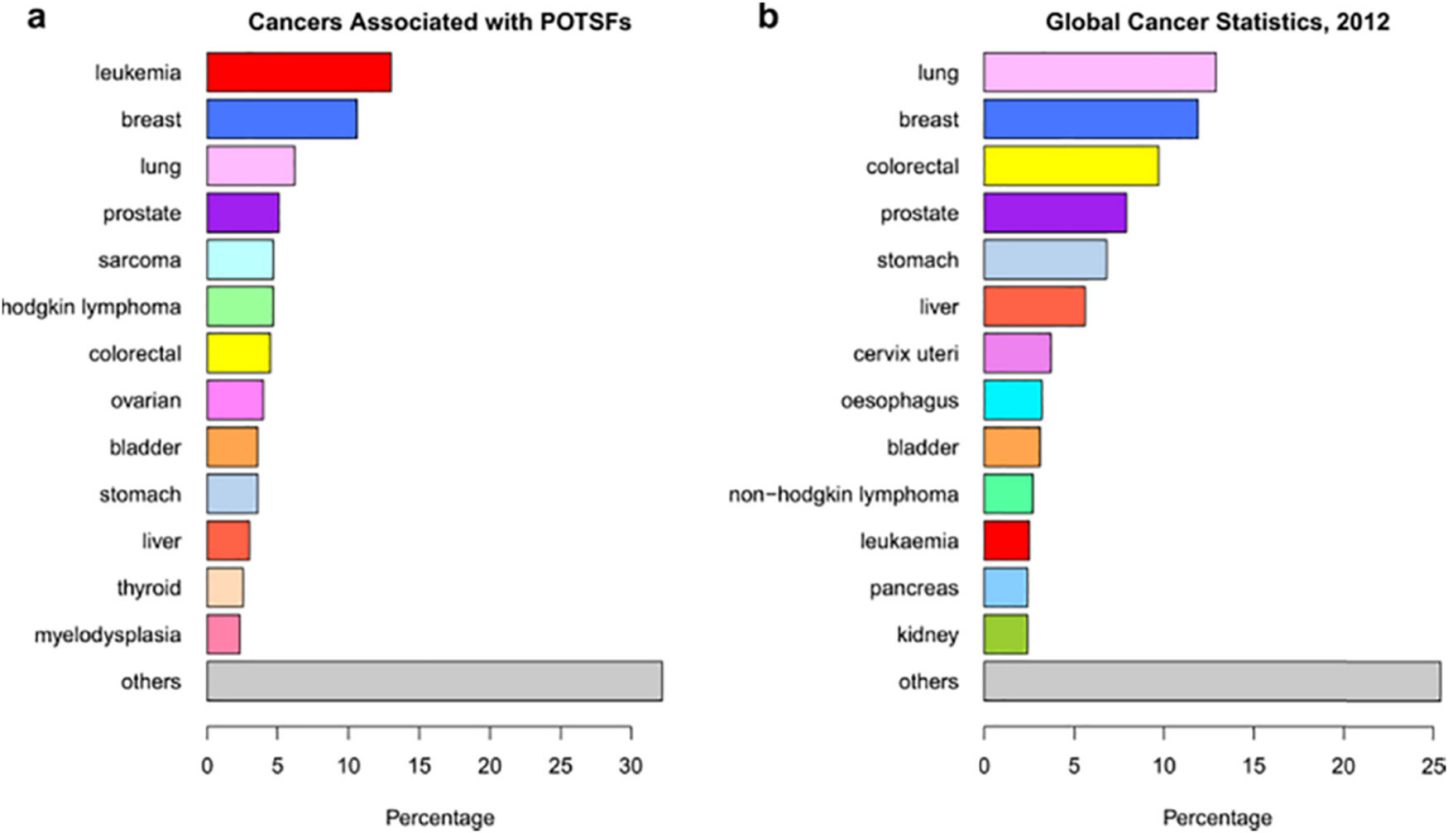
Global cancer statistics 2012. (a) The cancer types’ percentages associated with POTSFs. (b) The cancer types’ percentages documented in statistics of 2012’s global cancer. The same cancer type is marked with the same color in (a) and (b); e.g., lung is represented with pink color in both (a) and (b).

The data were processed by SPSS version 15.0 (SPSS Inc., Chicago, IL, USA). Continuous data were presented as mean ± SD and analyzed using Student’s *t*-test. Two-sided *P* values <0.05 were considered significant (Figure 1).

## 3 Results

### 3.1 Validation of Prox1 overexpressing A549 and H446 cell models

**Figure 2:**
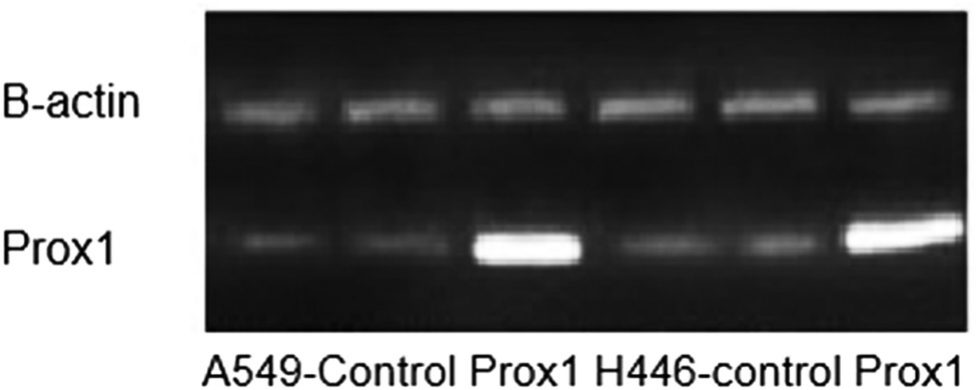
Agarose gel experiment to verify the expression of Prox1 in lung cancer.

**Figure 3:**
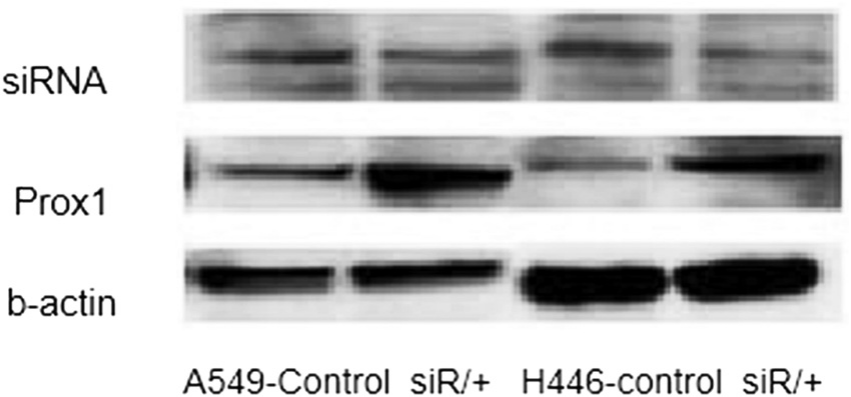
Expression of Prox1 protein in different lung cancer cell lines, including A549 and H446.

Prior to investigate the effect of Prox1 on the migration and invasion of lung cancer cells, we firstly confirmed the expression of Prox1 in A549 and H446 cells overexpressing Prox1 after plasmid transfection. According to the data from agarose gel experiment and western blot experiment, taking wild-type untransfected A549 and H446 cells as a control group, the expression of Prox1 in A549 and H446 cell groups overexpressing Prox1 after the introduction of transfected plasmid was significantly higher than that of wild-type untransfected A549 and H446 cells, while the A549 and H446 cell groups with low expression of Prox1 after transfection of siRNA were significantly lower than those of wild-type untransfected A549 and H446 cells. The experimental data showed that the model was successful. The experimental results are shown in Figures 2 and 3.

### 3.2 Growth of Prox1 overexpressing A549 and H446 cells

#### 3.2.1 Colony formation rate of Prox1 overexpressing A549 and H446 cells

**Figure 4:**
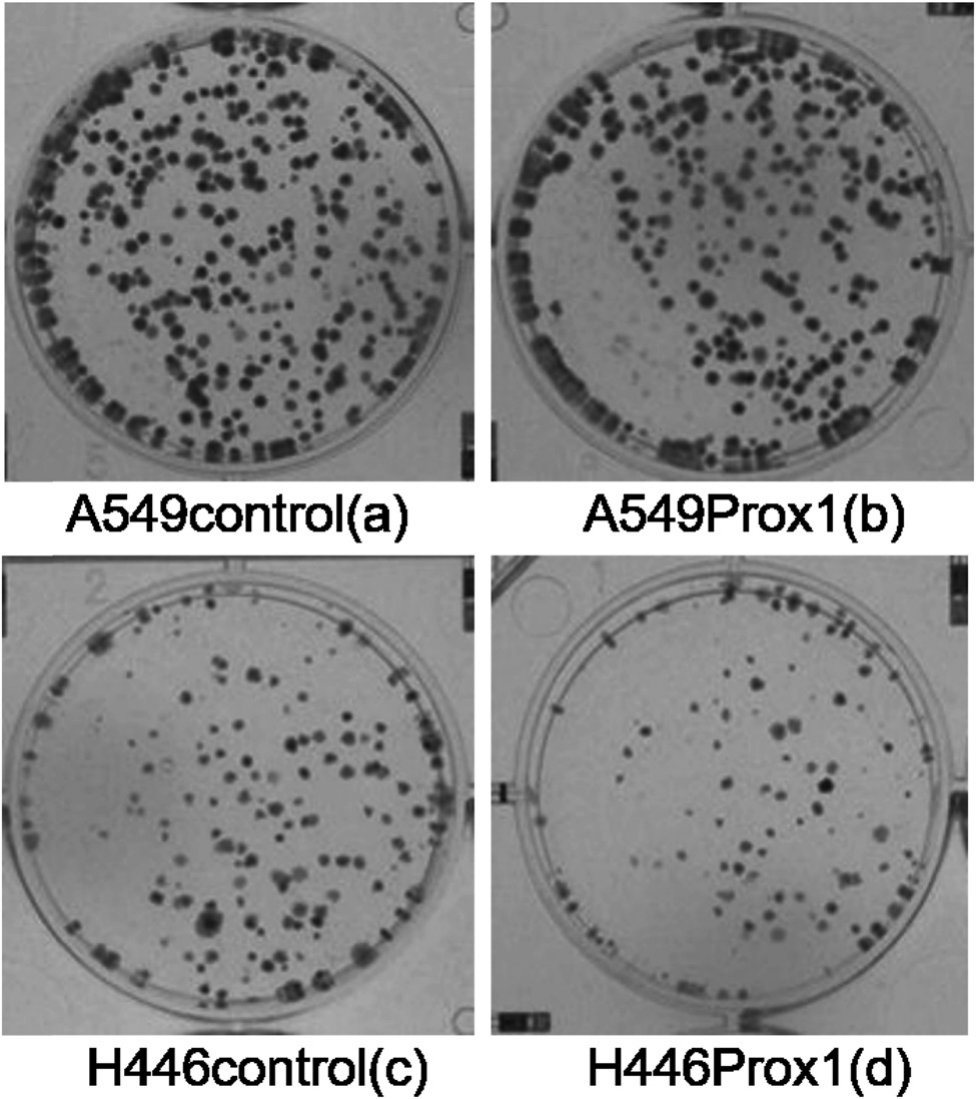
Plate colony formation assay to detect the effect of Prox1 plasmid transfection on the proliferation of A549 and H446 cells. Plate cloning was used to detect the clone formation of A549 and H446 cells overexpressing Prox1 after plasmid transfection and wild-type untransfected A549 and H446 cells (b and d). Wild-type untransfected A549 and H446 cells served as a control group (a and c).

Colony-forming efficiency reflects two important characteristics of cell population dependence and proliferation ability, that is to say, the cells that form clones must be adherent and have proliferation activity [17,18]. Therefore, this study used the method of plate cloning to detect the clone formation of A549 and H446 cells overexpressing Prox1 after plasmid transfection and wild-type untransfected A549 and H446 cells. The experimental results are shown in Figure 4. Taking wild-type untransfected A549 and H446 cells as a control group, the colony formation ability of A549 and H446 cells overexpressing Prox1 after plasmid transfection was significantly more potent than that of wild-type untransfected A549 and H446 cells. The results suggested that the expression of Prox1 could promote the growth of lung cancer cells.

#### 3.2.2 Cell growth curves of Prox1 overexpressing A549 and H446 cells

**Figure 5:**
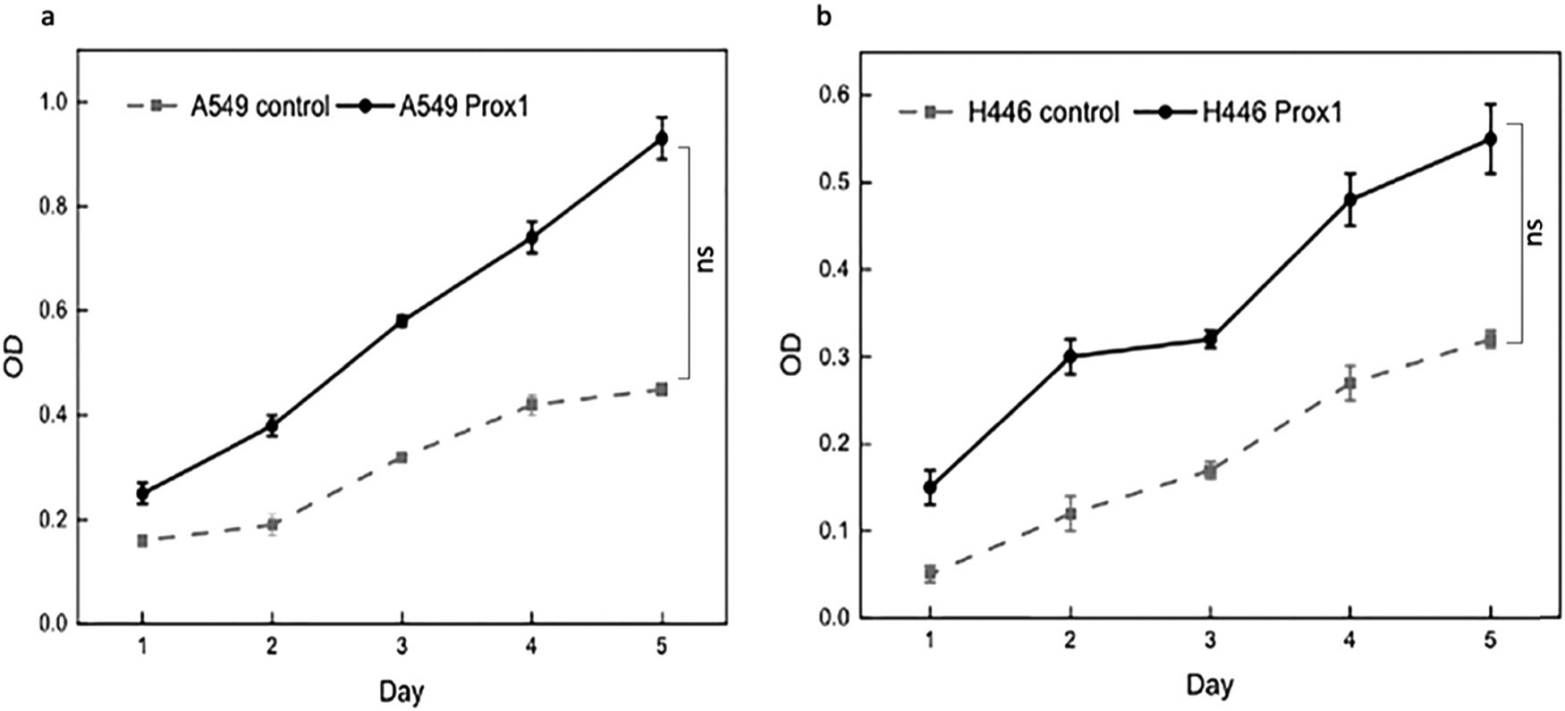
MTT assay to detect the growth curve of (a) A549 and (b) H446 cells after transfection with Prox1 plasmid. Data were presented as mean ± SD and analyzed using Student’s *t*-test (*n* = 3). *P* value; ns, no significance.

Relevant studies show that Prox1 expression is related to tumor stage and grade, suggesting that Prox1 participates in the proliferation process of lung cancer cells [19,20]. Therefore, the MTT method was used to detect A549 and H446 cells’ growth overexpressing Prox1 after plasmid transfection. As shown in Figure 5, the proliferation ability of A549 and H446 cells overexpressing Prox1 after plasmid transfection was significantly stronger than that of the control group with wild-type untransfected A549 and H446 cells. With the prolongation of culture time, the difference in cell proliferation ability was more obvious. The experimental results suggested that the increased expression of Prox1 could promote the growth of lung cancer cells.

### 3.3 Relationship between Prox1 and metastasis and invasion of lung cancer

#### 3.3.1 Effect of Prox1 expression on migration ability of lung cancer cells

**Figure 6:**
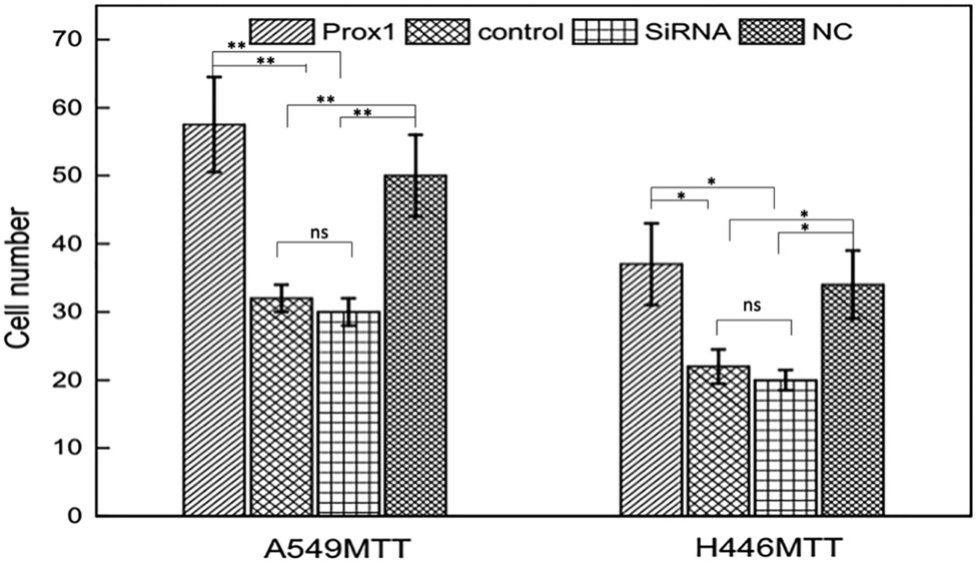
Transwell to detect the change of migration ability of A549 and H446 cells after changes in Prox1 expression. Wild-type untransfected A549 and H446 cells served the control group (*n* = 3). Data were presented as mean ± SD and analyzed using Student’s *t*-test (*n* = 3). ns, no significance; **P < 0.01; *P < 0.05.

To study the effect of Prox1 on the migration of lung cancer cells, Transwell experiment was used to detect the changes in migration ability of A549 and H446 cells overexpressing Prox1 after plasmid transfection and A549 and H446 cells with low expression of Prox1 after transfection of siRNA. The experimental results are shown in Figure 6. Taking wild-type untransfected A549 and H446 cells as the control group, the migration ability of A549 and H446 cells overexpressing Prox1 after plasmid transfection was stronger than that of wild-type untransfected A549 and H446 cells, while the migration ability of A549 and H446 cells with low expression of Prox1 after transfection of siRNA plasmid was lower than that of wild-type untransfected A549 and H446 cells. The results showed that the cell migration ability increased with the increased expression of Prox1 and decreased with the decreased expression of Prox1 (*P* < 0.01).

#### 3.3.2 Effect of Prox1 expression on invasive ability of lung cancer cells

**Figure 7:**
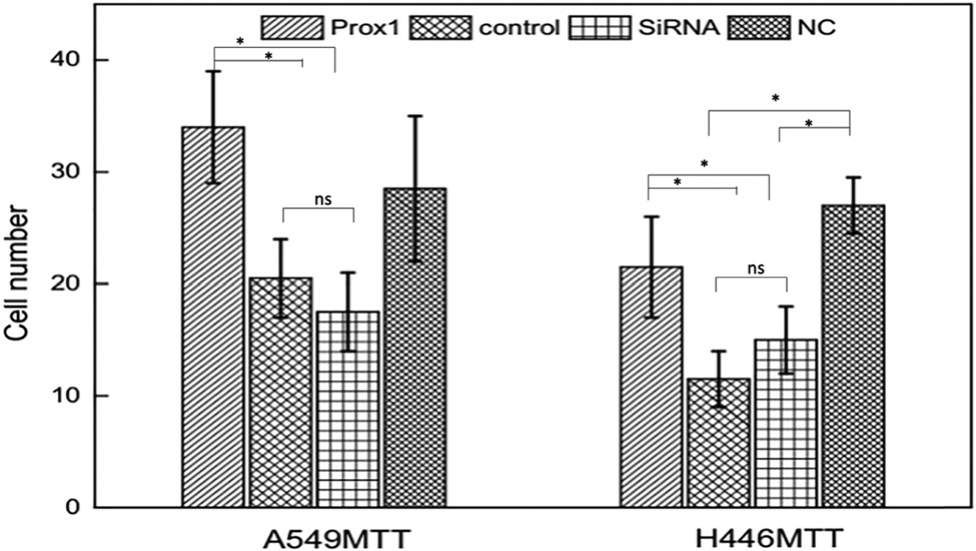
Transwell to detect the change of invasive ability of A549 and H446 cells after changes in Prox1 expression. Wild-type untransfected A549 and H446 cells served as the control group. Cell invasive ability increased with the increased expression of Prox1 and decreased with the decreased expression of Prox1 (*P* < 0.01). Data were presented as mean ± SD and analyzed using Student’s *t*-test (*n* = 3). ns, no significance; **P < 0.01; *P < 0.05.

Similarly, to study the effect of Prox1 on the invasive ability of A549 and H446 lung cancer cells, this study used Transwell experiment to detect the change of the invasive ability of A549 and H446 cells overexpressing Prox1 after plasmid transfection and A549 and H446 cells with low expression of Prox1 after transfection of siRNA. The experimental results are shown in Figure 7. Taking wild-type untransfected A549 and H446 cells as the control group, the invasive ability of A549 and H446 cells overexpressing Prox1 after plasmid transfection was stronger than that of wild-type untransfected A549 and H446 cells, while the invasive ability of A549 and H446 cells with low expression of Prox1 after transfection of siRNA plasmid was lower than that of wild-type untransfected A549 and H446 cells. The results showed that the invasive cell ability increased with the increased expression of Prox1 and decreased with the decreased expression of Prox1 (*P* < 0.01).

### 3.4 Effect of Prox1 on the expression of family

**Figure 8:**
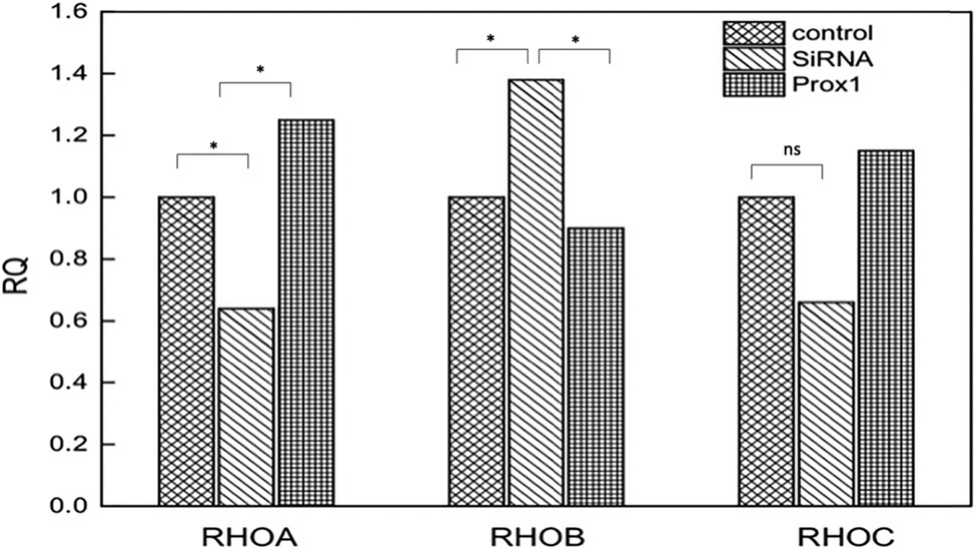
RT-PCR to detect RhoA, RhoB, and RhoC expression after changes in Prox1 expression. The expression of RhoA, RhoB, and RhoC in A549, H446 lung cancer cells was detected by transfection with plasmid Prox1 and interfering siRNA. When the Prox1 increased, the RhoA and RhoC increased, while RhoB decreased (*P* < 0.05); when Prox1 decreased, the RhoA and RhoC decreased, while RhoB increased (*P* > 0.05). ns, no significance; *P < 0.05, (*n* = 3).

**Figure 9:**
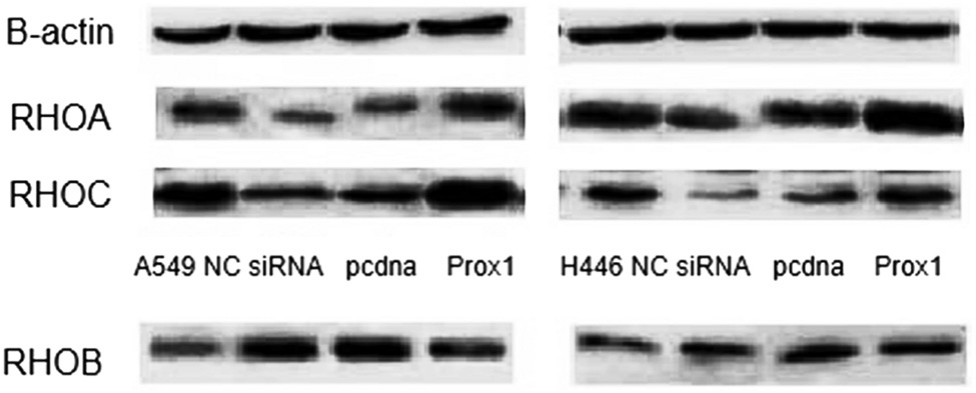
Western blot to detect RhoA, RhoB, and RhoC expression after changes in Prox1 expression. The expression of RhoA, RhoB, and RhoC in A549, H446 lung cancer cells was detected by transfection with plasmid Prox1 and interfering siRNA.

Since Rho family proteins can regulate cytoskeleton and play a certain role in the adhesion, invasion, and migration of tumor cells [21–24], this study speculated whether Prox1 acted through Rho family proteins. Therefore, western blot and real-time PCR analyses were used to detect the expression of RhoA, RhoB, and RhoC in A549, H446 lung cancer cells transfected with plasmid Prox1 and interfering siRNA. The experimental results are shown in Figure 8. Real-time PCR data showed that when Prox1 increased, RhoA and RhoC increased, while RhoB decreased (*P* < 0.05); when Prox1 decreased, RhoA and RhoC decreased, while RhoB increased (*P* > 0.05), followed by western blot experimental results shown in Figure 9. As the expression of Prox1 increased, the expression of RhoA and RhoC increased accordingly. In summary, after transfecting Prox1 plasmid in A549 and H446 cells with overexpression of Prox1 and interference of siRNA with Prox1, the change in Prox1 expression content was negatively correlated with the change in RhoB expression, while positively correlated with the changes in RhoA and RhoC expression. That is, when the content of Prox1 expression increased, RhoB expression decreased correspondingly, while RhoA and RhoC expression increased correspondingly. When Prox1 expression decreased, RhoB expression increased correspondingly, while RhoA and RhoC expression decreased correspondingly.

## 4 Discussion

Human Prox1 gene is located on chromosome 1q32.2–1q32.3, with a length of about 58 kb, contains at least five exons, and encodes 83 kD protein [25,26]. Prox1 plays a key role in the development of normal embryonic lens, liver, and neurons, especially in the nervous system and lens [25,26]. Related studies have shown that the expression of Prox1 in hepatocellular carcinoma is related to the degree of tumor differentiation [27]. Liver tumors with high Prox1 expression have a poor prognosis [28], and cancer cells’ proliferation is significantly accelerated [29]. It’s been suggested, that Prox1 is a downstream target gene of β-catenin/TCF in colon cancer, and Prox1 can lead to enhanced cell adhesion [30]. Others have confirmed that deletion, mutation, and hyper-methylation of the Prox1 gene can lead to the occurrence of biliary tumors, esophagus cancer, and breast cancer [20,31]. Previous experiments have proved that Rho protein can not only directly affect the construction of cytoskeleton but also participate in gene transcription regulation [32], thus indirectly affecting cytoskeleton and movement. The most important in RhoA-mediated malignant transformation is to regulate Stat3 activity. RhoB inhibits TGFβ receptor by reducing the binding of transcription factor AP1 to its promoter. RhoA and Rac1 can also regulate the degradation and reconstruction of the extracellular matrix by regulating the levels of matrix metalloproteinase and tissue inhibitor of metalloproteinase [33–36].

In this study, lung cancer A549 and H446 cells were transfected with Prox1NAD and siRNA plasmids, respectively, so that cells transfected with Prox1NAD plasmids overexpressed Prox1 and cells transfected with siRNA had low expression of Prox1. A series of studies and analyses were carried out on A549 and H446 cells after transfection. The results showed that taking untransfected wild-type A549 and H446 as blank controls, the expression level of Prox1mRNA and protein in A549 and H446 cells overexpressing Prox1 after plasmid transfection was high, while the expression level of Prox1mRNA and protein in A549 and H446 cells with low expression of Prox1 after siRNA transfection was low. The proliferation ability of A549 and H446 cells overexpressing Prox1 after plasmid transfection increased, and the number of cell invasion and migration also increased. The expression of RhoA and RhoC increased with the increase of Prox1 expression, while the expression of RhoB decreased relatively. So, the expression of Prox1 was related to the proliferation, migration, and invasion of lung cancer cells.

## 5 Conclusion

To sum up, the overexpression of Prox1 can promote the proliferation, migration, and invasion ability of lung cancer cells. It has also been found that the expression level of Prox1 is related to the expression level of Rho protein. Some studies have shown that Rho protein is not only involved in tumor invasion and metastasis but also in cycle regulation. Rho protein expression was positively correlated with Cyclin D1 expression. Therefore, Prox1 can regulate the proliferation of lung cancer cells by binding to Cyclin D1. Despite the strong proof of concept, the study needs to further investigate the role of Prox1 in the proliferation, migration, and invasion of lung cancer cells, and the role between Rho protein and Cyclin D1 need to be further demonstrated. Moreover, the role of Prox1 also need to be examined in an appropriate lung cancer animal model to get a more in-depth insight.

## References

[1] Chen W, Zheng R, Baade PD, Zhang S, Zeng H, Bray F, et al. Cancer statistics in China, 2015. CA Cancer J Clin. 2016;66(2):115–32. 10.3322/caac.21338 26808342

[2] Agnihotri NS, Astekar M. The role of novel prognostic markers PROX1 and FOXC2 in carcinogenesis of oral squamous cell carcinoma. J Exp Ther Oncol. 2018;12(3):171–84. 29790306

[3] Zeng H, Zheng R, Guo Y, Zhang S, Zou X, Wang N, et al. Cancer survival in China, 2003–2005: a population-based study. Int J Cancer. 2015;136(8):1921–30. 10.1002/ijc.29227 25242378

[4] Torre LA, Siegel RL, Jemal A. Lung cancer statistics. Adv Exp Med Biol. 2016;893:1–19. 10.1007/978-3-319-24223-1_1 26667336

[5] Gao T, Ma C, Li Y, Ju J, Kang X, Cai Y, et al. High expression of prospero-related homeobox-1 (Prox1) is associated with poor prognosis in patients with salivary adenoid cystic carcinoma. J Oral Maxillofac Surg. 2018;76(7):1440–6. 10.1016/j.joms.2017.12.032 29406257

[6] Kwak EL, Bang YJ, Camidge DR, Shaw AT, Solomon B, Maki RG, et al. Anaplastic lymphoma kinase inhibition in non-small-cell lung cancer. N Engl J Med. 2010;363(18):1693–703. 10.1056/NEJMoa1006448 PMC3014291 20979469

[7] Miettinen OS, Yankelevitz DF, Henschke CI. Screening for lung cancer. N Engl J Med. 2001;344(12):935 (author reply 936). 11263430

[8] Zheng R, Zeng H, Zuo T, Zhang S, Qiao Y, Zhou Q, et al. Lung cancer incidence and mortality in China, 2011. Thorac Cancer. 2016;7(1):94–9. 10.1111/1759-7714.12286 PMC4718125 26816543

[9] Herbst RS, Baas P, Kim DW, Felip E, Pérez-Gracia JL, Han JY, et al. Pembrolizumab versus docetaxel for previously treated, PD-L1-positive, advanced non-small-cell lung cancer (KEYNOTE-010): a randomised controlled trial. Lancet. 2016;387(10027):1540–50. 10.1016/S0140-6736(15)01281-7 26712084

[10] Luo D, Hu SY, Liu GX. Multi-channel promotion of lung cancer progress by bone marrow derived mesenchymal stem cells in tumor microenvironment. Zhonghua Zhong Liu Za Zhi. 2018;40(2):85–91. 10.3760/cma.j.issn.0253-3766.2018.02.002 29502366

[11] Chen X, Mao G, Chen H, Liu S, Wang S, Li X, et al. TW37 enhances the pro-apoptosis and anti-migration ability of gefitinib in Non-Small Cell Lung Cancer. Cell Mol Biol (Noisy-le-grand). 2018;64(4):6–10. 29631678

[12] Elsir T, Smits A, Lindström MS, Nistér M. Transcription factor PROX1: its role in development and cancer. Cancer Meta Rev. 2012;31(3–4):793–805. 10.1007/s10555-012-9390-8 22733308

[13] Zhu SH, Shan CJ, Wu ZF, Xu SZ. Proliferation of small cell lung cancer cell line reduced by knocking-down PROX1 via shRNA in lentivirus. Anticancer Res. 2013;33(8):3169–75. 23898075

[14] Lu M-H, Huang CC, Pan MR, Chen HH, Hung WC. Prospero homeobox 1 promotes epithelial–mesenchymal transition in colon cancer cells by inhibiting E-cadherin via miR-9. Clin Cancer Res. 2012;18(23):6416–25. 10.1158/1078-0432.CCR-12-0832 23045246

[15] Yokobori T, Bao P, Fukuchi M, Altan B, Ozawa D, Rokudai S, et al. Nuclear PROX1 is associated with hypoxia-inducible factor 1α expression and cancer progression in esophageal squamous cell carcinoma. Ann Surg Oncol. 2015;22(3):1566–73. 10.1245/s10434-015-4831-6 26310281

[16] Dudas J, Papoutsi M, Hecht M, Elmaouhoub A, Saile B, Christ B, et al. The homeobox transcription factor Prox1 is highly conserved in embryonic hepatoblasts and in adult and transformed hepatocytes, but is absent from bile duct epithelium. Anat Embryol (Berl). 2004;208(5):359–66. 10.1007/s00429-004-0403-4 15232737

[17] Skog M, Bono P, Lundin M, Lundin J, Louhimo J, Linder N, et al. Expression and prognostic value of transcription factor PROX1 in colorectal cancer. Br J Cancer. 2011;105(9):1346–51. 10.1038/bjc.2011.297 PMC3241535 21970873

[18] Papoutsi M, Dudas J, Becker J, Tripodi M, Opitz L, Ramadori G, et al. Gene regulation by homeobox transcription factor Prox1 in murine hepatoblasts. Cell Tissue Res. 2007;330(2):209–20. 10.1007/s00441-007-0477-4 17828556

[19] Yang J, Balbo S, Villalta PW, Hecht SS. Analysis of acrolein-derived 1, N(2)-propanodeoxyguanosine adducts in human lung DNA from smokers and nonsmokers. Chem Res Toxicol. 2019;32(2):318–25. 10.1021/acs.chemrestox.8b00326 PMC6644703 30644728

[20] Becker J, Wang B, Pavlakovic H, Buttler K, Wilting J. Homeobox transcription factor Prox1 in sympathetic ganglia of vertebrate embryos: correlation with human stage 4s neuroblastoma. Pediatr Res. 2010;68(2):112–7. 10.1203/PDR.0b013e3181e5bc0f 20453716

[21] Little AC, Danyal K, Bauer RA, Heppner DE, Hristova M, Dustin C, et al. Abstract 1681: DUOX1 expression in lung cancer disrupts pro-oncogenic activation mechanisms and localization of Src and EGFR. Vol. 76. Philadelphia, PA: AACR; 2016. p. 1681–1.

[22] Zhang B, Ji S, Ma F, Ma Q, Lu X, Chen X. miR-489 acts as a tumor suppressor in human gastric cancer by targeting PROX1. Am J Cancer Res. 2016;6(9):2021–30. PMC5043111 27725907

[23] Nguyen-Vu T, Wang J, Mesmar F, Mukhopadhyay S, Saxena A, McCollum CW, et al. Estrogen receptor beta reduces colon cancer metastasis through a novel miR-205 - PROX1 mechanism. Oncotarget. 2016;7(27):42159–71. 10.18632/oncotarget.9895 PMC5173124 27283988

[24] Joshi B, Strugnell SS, Goetz JG, Kojic LD, Cox ME, Griffith OL, et al. Phosphorylated caveolin-1 regulates Rho/ROCK-dependent focal adhesion dynamics and tumor cell migration and invasion. Cancer Res. 2008;68(20):8210–20. 10.1158/0008-5472.CAN-08-0343 18922892

[25] Makrodouli E, Oikonomou E, Koc M, Andera L, Sasazuki T, Shirasawa S, et al. BRAF and RAS oncogenes regulate Rho GTPase pathways to mediate migration and invasion properties in human colon cancer cells: a comparative study. Mol Cancer. 2011;10:118. 10.1186/1476-4598-10-118 PMC3189908 21943101

[26] Ishii J, Yazawa T, Chiba T, Shishido-Hara Y, Arimasu Y, Sato H, et al. PROX1 promotes secretory granule formation in medullary thyroid cancer cells. Endocrinology. 2016;157(3):1289–98. 10.1210/en.2015-1973 26760117

[27] Liu Y, Ye X, Zhang JB, Ouyang H, Shen Z, Wu Y, et al. PROX1 promotes hepatocellular carcinoma proliferation and sorafenib resistance by enhancing β-catenin expression and nuclear translocation. Oncogene. 2015;34(44):5524–35. 10.1038/onc.2015.7 25684142

[28] Dudas J, Mansuroglu T, Moriconi F, Haller F, Wilting J, Lorf T, et al. Altered regulation of Prox1-gene-expression in liver tumors. BMC Cancer. 2008;8:92–2. 10.1186/1471-2407-8-92 PMC2359759 18400094

[29] Liu Y, Zhang JB, Qin Y, Wang W, Wei L, Teng Y, et al. PROX1 promotes hepatocellular carcinoma metastasis by way of up-regulating hypoxia-inducible factor 1α expression and protein stability. Hepatology (Baltimore, MD). 2013;58(2):692–705. 10.1002/hep.26398 23505027

[30] Becker J, Wang B, Pavlakovic H, Buttler K, Wilting J. Homeobox transcription factor Prox1 in sympathetic ganglia of vertebrate embryos: correlation with human stage 4s neuroblastoma. Pediatric Res. 2010;68(2):112–7. 10.1203/PDR.0b013e3181e5bc0f 20453716

[31] Roodakker KR, Elsir T, Edqvist PD, Hägerstrand D, Carlson J, Lysiak M, et al. PROX1 is a novel pathway-specific prognostic biomarker for high-grade astrocytomas; results from independent glioblastoma cohorts stratified by age and IDH mutation status. Oncotarget. 2016;7(45):72431–42. 10.18632/oncotarget.11957 PMC5341919 27626492

[32] Rajakylä EK, Vartiainen MK. Rho, nuclear actin, and actin-binding proteins in the regulation of transcription and gene expression. Small GTPases. 2014;5:e27539. 10.4161/sgtp.27539 PMC4114923 24603113

[33] Miyazaki H, Yoshimatsu Y, Akatsu Y, Mishima K, Fukayama M, Watabe T, et al. Expression of platelet-derived growth factor receptor beta is maintained by Prox1 in lymphatic endothelial cells and is required for tumor lymphangiogenesis. Cancer Sci. 2014;105(9):1116–23. 10.1111/cas.12476 PMC4462385 24981766

[34] Jung E, Gardner D, Choi D, Park E, Jin Seong Y, Yang S, et al. Development and characterization of a novel Prox1-EGFP lymphatic and Schlemm’s canal reporter rat. Sci Rep. 2017;7(1):5577. 10.1038/s41598-017-06031-3 PMC5514086 28717161

[35] Shin HJ, Rho SB, Jung DC, Han IO, Oh ES, Kim JY. Carbonic anhydrase IX (CA9) modulates tumor-associated cell migration and invasion. J Cell Sci. 2011;124(Pt 7):1077–87. 10.1242/jcs.072207 21363891

[36] Cheng L, Zhou R, Chen M, Feng L, Li H. MicroRNA-150 targets Rho-associated protein kinase 1 to inhibit cell proliferation, migration and invasion in papillary thyroid carcinoma. Mol Med Rep. 2017;16(2):2217–24. 10.3892/mmr.2017.6842 28656254

